# Epidemiology, Treatment Trends, and Outcomes of Multiple Myeloma in the Middle East and Africa: A Systematic Review

**DOI:** 10.46989/001c.92555

**Published:** 2024-02-22

**Authors:** Mervat Mattar, Ali Bazarbachi, Omar Abduljalil, Bassam Francis, Arif Alam, Vivian Blunk

**Affiliations:** 1 Dept of Internal Medicine Hematology Unit, Cairo University, Cairo, Egypt; 2 Dept of Medicine, Hematology and Oncology American University of Beirut, Beirut, Lebanon; 3 Dept of Hematology & Stem Cell Transplant King Fahad Specialist Hospital, Dammam, Saudi Arabi; 4 Hematology Unit and Bone Marrow Transplant Center Baghdad teaching Hospital, Baghdad, Iraq; 5 Dept of Hematology Oncology Tawan Hospital, Abu Dhabi, United Arab Emirates; 6 Oncology Emerging Markets Pfizer, São Paulo, Brazil

**Keywords:** Multiple myeloma, incidence, mortality, epidemiology, Middle East

## Abstract

**Background:**

Globally, multiple myeloma (MM) ranks 24^th^ among the most common cancers. The Middle East and Africa are affected by an increasing trend in MM incidence, owing to several underlying factors. This systematic review aims to assess the epidemiology, patient characteristics, and treatment outcomes associated with MM in selected countries in the Middle East and Africa.

**Methods:**

An electronic search was performed in the PubMed/MEDLINE database. Abstracts presented at the annual meetings of the American Society of Clinical Oncology, American Society of Hematology, and European Society for Medical Oncology and the GLOBOCAN registry were searched. Qualitative analysis was performed.

**Results:**

A total of 412 articles were screened, and 14 were selected. The five-year prevalence per 100,000 gathered from country-wise GLOBOCAN data ranged between 155 in Kuwait and 5,625 in North Africa. The identified treatment options were proteasome inhibitors such as bortezomib, drugs such as thalidomide, lenalidomide, dexamethasone, melphalan, and cyclophosphamide, and newer drugs such as daratumumab.

**Conclusion:**

Improved diagnostic capability has increased the incidence of MM in this region. However, advanced drugs and treatment regimens remain unaffordable in many countries of these regions. Therefore, understanding the trends of the disease and improving healthcare settings are imperative.

## INTRODUCTION

Multiple myeloma (MM) is a hematological malignancy characterized by abnormal clonal proliferation of plasma cells within the bone marrow, progressively leading to end-organ damage. The condition characteristically evolves through a spectrum, starting with a precursor state of monoclonal gammopathy of undetermined significance and progressing to Smoldering MM (SMM), which eventually develops into symptomatic MM.^1,2^ A unique feature of MM is its varying genomic landscape that results in intra-tumoral, spatial, and temporal heterogeneity, which leads to heterogeneity in treatment outcomes and the subsequent evolution of resistance to treatments.^2,3^

Globally, MM is ranked 24^th^ among the most common cancers, and accounts for 1% of all cancers.^4^ It is the second most common hematologic malignancy after lymphoma, and accounts for approximately 10–17% of all hematologic malignancies.^1,5^ The number of MM cases has increased more than two times over the last three decades, with its incidence rising from 65,940 (95% uncertainty interval (UI) = 155,688–74,058) in 1990 to 155,688 (95% UI = 136,585–172,577) in 2019.^4^ Although MM occurs across all races and locations, its incidence varies among different ethnicities.

Treatments for myeloma have improved substantially over the years owing to the advancement in autologous stem cell transplantation (ASCT) in the 1990s and the development of novel agents in the early 2000s. This has enhanced the estimated life expectancy of patients with MM from a 5-year relative survival rate of 35% in 2000 to above 50% recently. However, MM remains incurable. Although most cases can be managed with outpatient therapy, the condition is fatal, with most patients dying of the disease.^6,7^

Recent advances in treatment modalities have largely resulted in better outcomes for patients in high-income countries. In low- and middle-income countries (LMICs), receiving cancer care is often affected by factors such as a lack of access to general and specialised healthcare, advanced diagnostics, and treatments, late presentation, and inadequate treatment leading to poor outcomes.^6^ In addition to these challenges, some of the highest increases in cases of MM are seen in middle and lower-middle socio-demographic index (SDI) countries.^6^ The Middle East and Africa are specifically affected by an upward trend in MM incidence. In addition, the limited availability of resources and the socioeconomic burden of the disease are a matter of concern.^8^

Studies conducted in Africa indicate that MM constitutes 8.2% of all blood cancers in the region.^9^ However, various studies have found that the incidence of MM among African Americans in the United States was significantly higher than that of European Americans in the United States. Whether this difference is due to limited access to diagnostic technology in lower SDI countries or a reflection of differences in disease biology requires further research. Problems of proper access to effective care exist in many countries in sub-Saharan Africa. A notable issue is the paucity of stem cell transplant centers in those countries and the non-availability of drugs such as lenalidomide and bortezomib.^6^

The Middle East has also experienced an increase in the incidence of MM. The UAE and Qatar have reported the highest increases in MM cases across the world in the last three decades.^8^ In 2015, the Saudi Cancer Registry reported that MM represented one percent of all cancers and five percent of all hematological malignancies.^1^ Access to novel drugs and treatments are challenges in these countries as well. The lack of data affects the decision-making process and restricts treatment availability.^8^

Owing to the increasing burden of the disease, lack of cost-effective strategies, and insufficient literature in this regard, we aimed to gather and assess data from studies reporting treatment outcomes associated with MM from selected countries in the Middle East and Africa. Data on treatment regimens, modifications in existing regimens, patient characteristics, and factors affecting outcomes were collected and analysed. This study provides an overview of the available therapeutic options in use in these settings.

## METHODOLOGY

### Literature search

This systematic review was conducted in adherence to the Preferred Reporting Items for Systematic Reviews and Meta-Analyses (PRISMA) statement. The search method to identify relevant studies using focus questions is as follows:

Population: Adult patients (>18 years of age) diagnosed with MM; Intervention: Not applicable; Determinant: Prevalence and treatment regimens

### Objectives

**Primary objective:** To estimate the prevalence of newly diagnosed and refractory MM in the Middle Eastern and African countries of Saudi Arabia, UAE, Kuwait, Iraq, Algeria, Egypt, South Africa, Lebanon, Jordan, Tunisia, North Africa and Morocco.

**Secondary objectives**: 1) To characterise the distribution of MM and the various treatments/regimens being used in various subgroups of the populations in the Middle East and African regions; 2) To assess the demographic characteristics of patients with MM in the Middle East and African regions from 2010 to 2022.

### Eligibility criteria

This systematic review was limited to original research for each of the key research questions. Articles published in English and French were considered. The geographical settings were limited to selected countries in the Middle East and Africa, namely, Saudi Arabia, UAE, Kuwait, Iraq, Algeria, Egypt, South Africa, Lebanon, Morocco, Jordan, North Africa, and Tunisia.

### Definition of the disease

This systematic review uses data on adults diagnosed with MM. The International Statistical Classification of Diseases and Related Health Problems, Tenth Revision (ICD-10) codes MM as C90 (MM and malignant plasma cell neoplasms).^10^ Studies on patients with MM and concomitant amyloidosis, therapy-related MM, secondary MM, and SMM were not included.

### Information sources and search protocols

A detailed electronic search in the PubMed/MEDLINE database was performed. Relevant abstracts presented at the annual meetings of the American Society of Clinical Oncology (ASCO), American Society of Hematology (ASH), and European Society for Medical Oncology (ESMO) and published on the respective conference websites in the last 3 years were also included. The latest epidemiological data on MM from local resources (registries, if available) and GLOBOCAN were also acquired. Only studies published in English and French and conducted between 2010 and 2022 were included. There was no limit with respect to the ethnic status of the participants. The search strategy is provided in Appendix 1.

Two authors (MM and VB) independently deduplicated the gathered results and further examined the remaining articles by their title and abstract. Any disagreement was resolved with a third reviewer (AB). The full texts of the selected studies were obtained, further analysed for inclusion/exclusion criteria and reviewed thereafter.

The study protocol was registered in the International Prospective Register of Systematic Reviews (PROSPERO) database with reference number CRD42022339932.

### Data collection process and data items

A Microsoft Excel spreadsheet was used to classify the selected studies based on: 1) name of the first author; 2) title of the study; 3) reference details; 4) study centre, place, and region; 5) study objective(s); 6) study design; 7) study period; 8) overall description of the patient population; 9) number of patients with MM; 10) number of patients with newly-diagnosed MM/relapsed MM/refractory MM/triple-refractory MM; 11) age-wise distribution of patients; 12) gender-wise distribution of patients; 13) subtype-wise distribution of patients; 14) distribution of patients according to comorbidities; 15) incidence of MM; 16) prevalence of MM; 17) treatment pattern/regimen (induction, consolidation, and maintenance regimens in newly diagnosed MM cases, treatment of relapsed MM in first relapse and second or higher relapse MM cases, treatment regimen in transplant-ineligible MM patients); 18) key study conclusions; and 19) article link.

### Quality assessment

All the selected studies were independently and duplicately assessed by two of the authors (MM and VB) using the Joanna Briggs Institute (JBI) tool for critical appraisal of analytical studies. Each study was assessed in the following domains: criteria for inclusion; study subjects and study settings; exposure measurement; measurement of condition; confounding factors; strategies to handle confounding factors; outcome measurement; and appropriate statistical analysis.^11^ Each study was scored individually. To consolidate the scores, ‘not applicable’ was counted as a ‘yes’, whereas ‘unclear’ was counted as a ‘no’. The sum of the points awarded to each question was divided by the highest possible score (8) to generate a fraction (between 0 and 1). Scores of 0–0.3, 0.4–0.6, and 0.7–1.0 were considered to be of low, moderate, and high quality, respectively. The review authors were not blinded to author information and affiliation. A third reviewer (AB) resolved any disagreement.

## RESULTS

**Figure 1.**
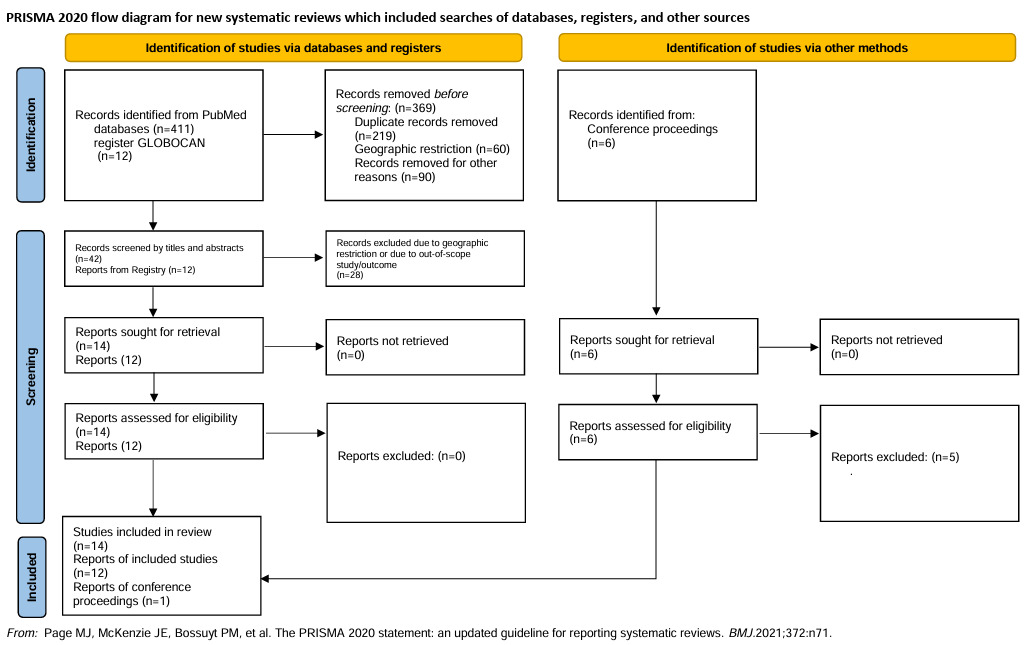
Flow diagram for study selection.^25^

The preliminary search through the PubMed database from 2010 till 13 June 2022 yielded 412 articles. Of these, 14 full-text articles were selected and retrieved for full-text analysis.^1,6,12–23^ The conference abstracts published in the annual meetings of the ASCO, ASH, and ESMO yielded six articles, of which one was selected.^24^ Additionally, the GLOBOCAN registry was searched for country-wise data, yielding 12 reports (Figure 1).

### Quality assessment

**Figure 2.**
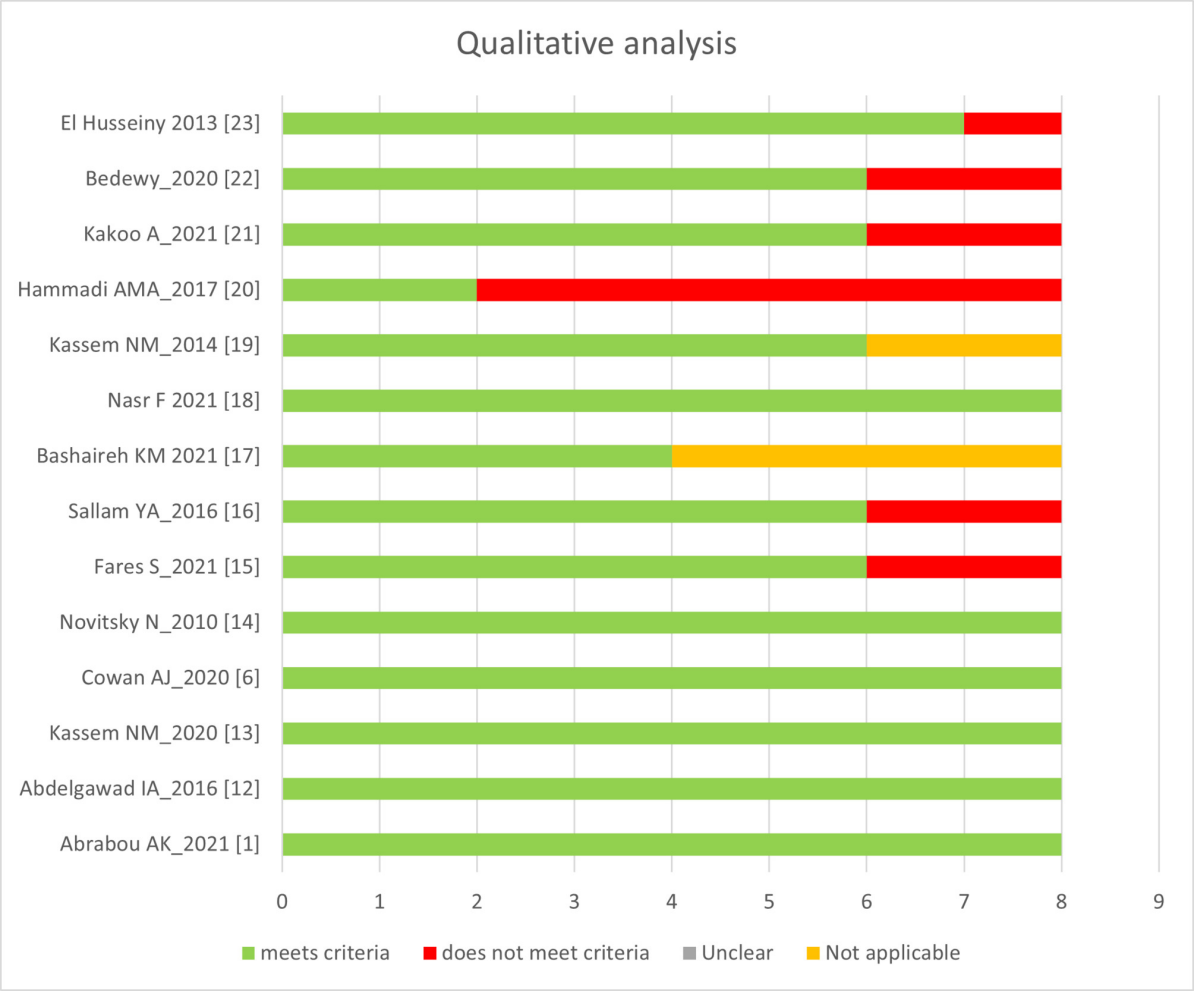
Qualitative analysis based on the Joanna Briggs Institute critical appraisal tool.

**Figure 3.**
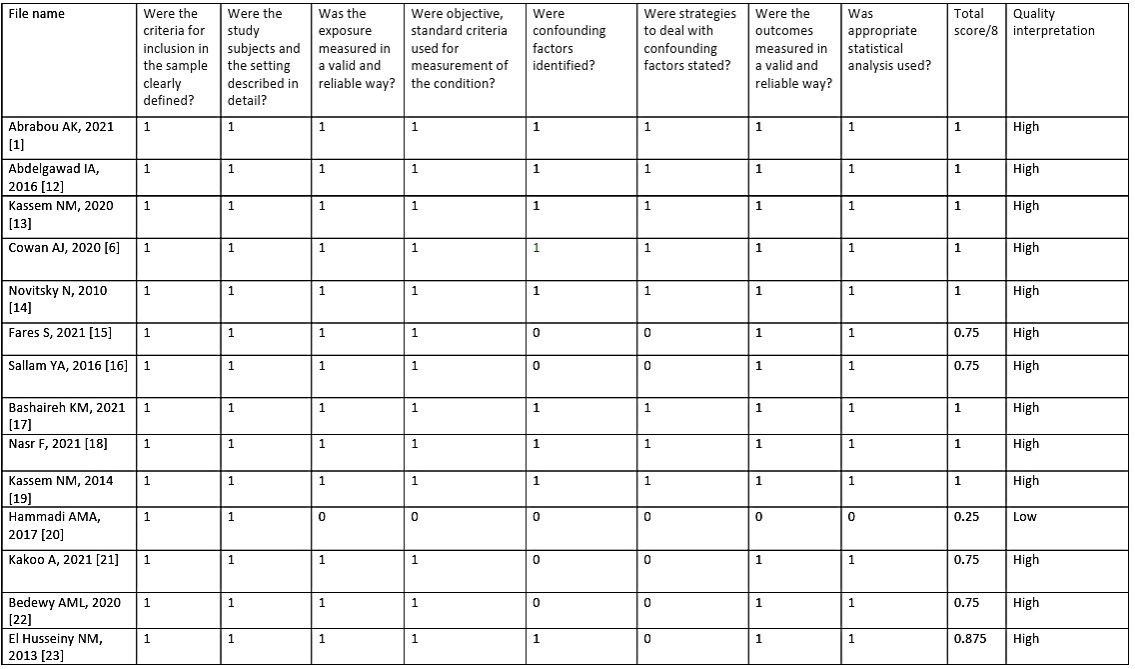
Consolidated quality assessment of included studies. 1 = yes/not applicable; 0= no/unclear.

Figure 2 is a representation of the qualitative assessment of the 14 included studies using the JBI critical appraisal tool.^11^ One study available from conference proceedings was excluded.^24^ Figure 3 provides a quality assessment of the included studies based on scoring criteria. One of the 14 included studies had low quality and was excluded from the synthesis of results.^20^

### Study design

The sample sizes in the included studies ranged between 33 to 1,010 patients. The median ages of the participants across the studies were between 40 and 63 years. The longest study duration was 156 months. The characteristics of the included studies are presented in Table 1 (provided as Appendix 2).

### Patient characteristics

The most common immunoglobulin found in patients with MM in the included studies was IgG, between 47.6% and 73%.^1,14,18,19,23^ Of the 10 studies in which patients were classified according to the International Staging System (ISS), five reported stage 3 (40.6%–53% of all MM cases)^1,12,18,21,24^ whereas six reported that MM was most commonly diagnosed at stage 2 (41%–67%).^13,16,19,22,23^ The male:female ratios ranged from 0.61 to 2.0 across all 14 reports.^14,24^ Eleven studies enrolled newly diagnosed patients with MM.^1,12–16,18,19,21–23^ In 6, conventional cytogenetics with fluorescence *in situ* hybridisation (FISH) to detect chromosomal abnormalities was performed.^1,16,18,19,23^ Cytogenetic analyses in a study by Kassem *et al.* (2014) showed that 30% of the MM tissue samples analysed had 13q deletion as assessed by FISH.^19^ Abdrabou *et al*. (2021) indicated that the most common genetic abnormality in MM tissues was the deletion of chromosome 13; whereas Fares *et al.* (2021) further reported genetic abnormalities such as translocation (t (11,14), t (4,14)) and deletion of 17p and 13q in MM tissues.

### Incidence, prevalence, and mortality

These were gathered from the GLOBOCAN 2020 registry of the respective countries and summarised in Table 2. The incidence per 100,000 in the included studies ranged between 58 in Kuwait and 2,535 in North Africa (inclusive of seven countries, namely Algeria, Egypt, Libya, Morocco, Sudan, Tunisia, and Western Sahara). The 5-year prevalence per 100,000 was the lowest in Kuwait, at 155, and the highest in North Africa, at 5,625. Mortality ranged between 33 in the UAE and 2,185 in North Africa.

The included studies did not provide epidemiological data. GLOBOCAN 2020 data are inclusive of epidemiological data from national and local sources in the respective countries, if available.

**Table 2.** Country-wise incidence, 5-year prevalence, and mortality^26–38^

**Country**	**Incidence/100,000**	**5-Year prevalence/100,000**	**Mortality/100,000**
Algeria	752	1813	613
Egypt	736	1658	649
Iraq	440	979	367
Jordan	165	389	127
Kuwait	58	155	37
Lebanon	205	477	169
Morocco	461	1003	389
North Africa	2535	5625	2185
Saudi Arabia	265	733	206
South Africa	1294	2931	975
Tunisia	187	443	155
UAE	60	173	33

### Summary of treatment outcomes

The treatment options identified in the included studies were proteasome inhibitors such as bortezomib, immunomodulatory drugs such as thalidomide and lenalidomide, corticosteroids such as dexamethasone, and alkylating agents such as melphalan and cyclophosphamide. Additionally, the use of daratumumab, a monoclonal antibody targeting CD38, has also been evaluated.

In a study conducted in Egypt, patients younger than 60 years received a combination therapy of Velcade (bortezomib), Revlimid (lenalidomide) and dexamethasone (VRD), whereas those aged 70 years and above received Endoxan–dexamethasone because bortezomib was not approved for such patients. They were further maintained with lenalidomide.^13^ A study conducted in Morocco concluded that in developing countries, ASCT with no cryopreservation reduces the costs of hospitalization and essential equipment.^15^

Novitzky *et al*. concluded that an intensified dose of melphalan is beneficial for treating younger patients. Moreover, the affordability of such novel agents is also of concern. In this context, the authors suggested that intensified melphalan provides a balance between matters of affordability, treatment effectiveness, and toxicity.^14^ The authors also compared the cost-effectiveness of melphalan therapy with newer agents and concluded that the overall expenditure with the melphalan strategy was US $3546.50 for an average of 10 patients, that of thalidomide was US $1387.61 for a 28-day course at a dose of 200 mg/day, and that of bortezomib was US $7379.59 per each of three cycles, indicating the cost-effectiveness of the melphalan strategy. A review by Esma *et al.* highlighted that melphalan remains a crucial, cost-effective, and beneficial therapy, and can be combined with novel agents to improve its efficacy.^39^

Kakoo *et al.* compared the impact of bortezomib versus daratumumab (DARA) on lymphocyte subpopulations in patients with MM. The authors concluded that bortezomib decreases the lymphocyte count for CD8 T cells, whereas DARA positively impacts the lymphocytes. Hence, DARA may be more specific than bortezomib when targeting myeloma cells.

Haleeqa *et al.* conducted a retrospective study involving patients diagnosed with MM in the UAE and reported that of the 25 eligible transplant patients who received ASCT, two died and the status of one patient remained unknown. The authors also mentioned that there was a lack of ASCT services in the UAE, and discussed the possibilities of improved results with the introduction of the same.

In comparison, a retrospective study by Alam *et al*. (2018) reported the use of bortezomib and immunomodulatory drug-based regimens in the UAE, where a median follow-up of 216 days led to a complete response (CR) + very good partial response (VGPR) of 72%. The lower CR+VGPR may be a consequence of patients seeking care outside the UAE, the use of a double instead of a triple regimen, and the absence of ASCT.^40^

Both studies^24,40^ have advocated for the introduction of ASCT services in the UAE for better prognosis in patients with MM.

### Critical factors affecting treatment outcomes

Poor prognosis was observed in MM patients with chromosome 13 deletion, with a hazard ratio (HR) of 6.22 (p<0.001) and 7.87 (p<0.001) in univariate and multivariate analyses, respectively.^1^ However, recent studies suggested that the survival rates among patients (with or without chromosome 13 deletion) who received bortezomib were similar, suggesting that bortezomib can overcome the adverse impact of chromosome 13 deletion.^41^

Younger age was associated with improved overall survival (OS) compared with patients aged more than 75 years (p<0.001).^19^ Older age (p<0.04) was associated with poor OS in Nivotzky *et al.*.^14^ All other studies reported no such significant difference.

Abnormal bilirubin levels and higher serum calcium levels were associated with higher HR of 7.04 and 10.9, respectively (with p-values of <0.001 and 0.038, respectively) for disease-free survival in MM.^13^ Similar findings were reported in the study by Nasr *et al*. (2021), in which the presence of hypercalcemia during diagnosis was associated with shorter progression-free survival (PFS) (unadjusted HR = 6.48; p<0.008) and OS (unadjusted HR = 6.58; p<0.006). In addition, this study also reported that MM patients with renal disease have shorter PFS times (unadjusted HR = 3.78; p<0.037).

Other factors that significantly impacted the survival of MM patients included lower (than the median) haemoglobin levels on presentation (p<0.01), lower Karnofsky scores (p<0.01), and older age (p<0.04).^14^ International staging system (ISS) stage III was associated with a worse prognosis in patients with MM^16^

### Prognostic indicators

Among MM patients, the levels of interleukin-10 (IL-10), B-cell activating factor, interleukin-6 (IL-6), and β2M in the serum were significantly higher compared with the normal control group (p<0.001).^12^ In addition, a significant reduction in the expression levels of the cancer inhibitor of protein phosphatase 2A (CIP2A) after treatment with bortezomib–dexamethasone therapy (BD) was associated with an improved prognosis for patients with MM. Patients with expression levels of CIP2A ≤16.45 EU were more likely to respond to BD therapy than those with expression levels of CIP2A >16.45 EU (p<0.005).^22^

### Treatment outcomes

Treatment outcomes included CR, partial response (PR), VGPR, PFS and OS. Wide variations and heterogeneity were noted among the included studies. The diversity of treatment regimens may explain the variations in CR. Sallam *et al.* (2016) reported a CR of 15.3% when the following treatments were administered at NCI, Cairo University: 34 patients received vincristine, adriamycin (doxorubicin), and Decadrone (VAD); 17 received melphalan and prednisone; and 3 received dexamethasone; radiotherapy was given to 56.7%, and bisphosphonates were given to 43.3% patients. However, a retrospective study conducted by Abdrabou *et al*. (2021) in Saudi Arabia reported a CR of >50% after induction therapy; the CR increased to 78.1% after ASCT (as per the IMWG 2014 response criteria). In that study, 28.4% of the patients were treated with VAD, 11.8% with the thalidomide-dexamethasone (TD) protocol, and 24.9% with Velcade-cyclophosphamide-dexamethasone (VCD).

VGPR varied from 11.3%^1^ in pre-transplant patients to 33%^14^ in those who were treated with submyeloablative doses of melphalan and received stem cell support. The smallest median values of PFS (6 months) were seen in patients who received VAD as first line of treatment,^16^ while the highest (75.11 months) were in patients who received transplants.^18^ The OS for a combination treatment of endoxan with dexamethasone was 5.1 months^13^; for post-transplant patients, the OS was reported as 202 months.^1^

## DISCUSSION

This review was aimed at estimating the prevalence of newly diagnosed and refractory MM cases in the Middle East and African countries, namely Saudi Arabia, UAE, Iraq, Algeria, Egypt, South Africa, Lebanon, Jordan, North Africa, Tunisia and Morocco, and characterising the various treatments/regimens provided in these regions. It was also aimed at assessing the demographic characteristics of patients with MM in the Middle East and Africa from 2010 to 2022.

The incidence, 5-year prevalence, and mortality of MM in South Africa were the highest among selected countries in Africa, whereas Iraq was the highest in terms of the said indicators in the Middle East. The prevalence of MM in the African population of the sub-Saharan region was relatively low; however, note that it is still increasing. Increasing life expectancy and aging population are plausible explanations for the same.^6^ Another contributing factor could be a low suspicion index among healthcare practitioners. This may be a result of limited access to diagnostic services which, in turn, may result in missed diagnosis. The affordability of the treatment itself could be a potential contributing factor. Countries in Africa are additionally facing the challenge of limited resources in terms of pathologists and cytotechnologists.^42^ Studies have also shown that when MM is treated in resource-rich settings, it is associated with improved outcomes. Nevertheless, healthcare mechanisms often remain insufficient, and services are offered to large populations, both urban and rural, only at centers in urban and economic settings.^9^

In a study by Ludwig et al. a poor survival outcome (measured as 1-mortality to incidence ratio (1-MIR), a proxy measure for 5-year survival rates) was reported. Factors such as access to cancer drugs, awareness or empowerment of the patient, healthcare access and quality index, and healthcare expenditures were associated with the 1-MIR. However, an exception to the above was observed in the UAE, a country with a high gross domestic product (GDP) but reporting a 1-MIR of only 15%.^43^ Additionally, the cancer country profile 2020 report suggested that public cancer care centres / 10,000 cancer patients in UAE (high income country) ratios were 6.4% while in Iraq (upper middle-income country) and Kuwait (high income country), they were 17.4% and 11.2%, respectively.^44–46^

A study conducted in 2017 in Algeria reported that the Algerian Multiple Myeloma Registry 2014, the first in the region, was a reliable data bank and an integral aspect of ensuring more diagnostic and prognostic assessment for the patients. Alam et al.^47^ reported the need to develop infrastructure for consistent testing and standardisation of diagnosis, treatment, and follow-up to improve care for patients in this region.^40^ A single centre study in Saudi Arabia reported that most of their MM patients were younger with a median age of 60 years and with a high ISS stage, but their response to therapy and outcome were good, suggesting the need to assess these observations in the future.^48^ These studies highlight the need for research in these regions, which facilitates the process of improving care and patient outcomes.

Articles included in this systematic review predominantly used the ISS classification for prognostic staging. The most common staging at the presentation was stage III, followed by stage II. Delays in referral and the need to travel longer distances to reach tertiary care centers could be plausible explanations for this trend.

In the Middle East, despite recent advancements in diagnosis and treatment options, there are several challenges, particularly with respect to relapsed cases. Although newer drugs and other therapeutic interventions improve the prognosis of MM, treatment accessibility remains limited across the region. The same applies to minimal residual disease (MRD) negativity evaluation. Its notable prognostic implications are indubitable; however, clear definitions of time to duration of MRD need to be established, in addition to the optimal method for MRD assessment. Moreover, evaluation techniques are costly and are not readily available across the region. Thus, the feasibility and cost-effectiveness of MRD assessment in daily practice remains to be further explored in the Middle East.^8^

In Lebanon, despite the recent guidelines shared by the Lebanese Society of Hematology and Blood Transfusion, the lack of comprehensive data hinders the informed decision-making process.^8^ There have been significant enhancements in the therapeutic modalities of MM in the last few years. The introduction of ASCT has positively impacted OS. In addition, novel agents such as proteasome inhibitors, immunomodulatory drugs, monoclonal antibodies, and CAR-T cells have improved OS and PFS. However, these novel drugs come with issues of affordability, and a cost-benefit balance must be considered.^49^ Despite this, the CAR-T cell therapy treatment market is expected to gain growth in the coming years in the Middle East and African regions.^50^ This study also explored the various treatment regimens specific to each centre and country. Accordingly, various CR, PR, and VGPR values were observed in this systematic review.

Several studies have presented their outcomes in terms of OS and disease-free progression (DFS). The OS (where data were available) ranged from 5.1 to 202 months. A combination of VAD, thalidomide–dexamethasone, bortezomib/thalidomide/dexamethasone and VCD was used. Following this, haematopoietic cell mobilisation, high-dose chemotherapy and ASCT were performed. The international standards followed by Abdrabou *et al.*, resulted in the highest OS of 202 months.^1^ The higher OS was probably related to the novel agents used and the inclusion of younger MM patients in the study. However, that report emphasised its limitations with respect to its retrospective nature and use of registry-level data.^1^

Some of the included studies focussed on cost-effective treatment strategies to enhance the survival rates of patients.^14,15^ In this region, where the economic burden of treating MM is a critical factor, such studies are helpful. The recent introduction of the generic version of lenalidomide into the global markets holds promise for greater affordability of essential drugs.^51^ Nonetheless, the need to bridge the gaps in affordability and availability of these drugs and regimens remains important.

The review does have a few limitations. The majority of the included studies were observational in nature. There was variation with respect to patient characteristics, regional differences, treatment patterns, use of novel drugs, and follow-up periods across the studies. Nevertheless, the review encompassed data from representative countries in the Middle East and Africa, providing a comprehensive depiction of the MM distribution and treatment trends.

## CONCLUSION

The incidence of MM is increasing in the Middle East and Africa. However, cost effectiveness analysis of novel drugs and treatment regimens and their impact on patients in this region remains to be performed. However, improving healthcare settings and understanding the trends of the disease in this region through in-depth country-specific research will pave the way for improved care.

### Competing Interests

Ali Bazarbachi: Speaker bureau or advisory board: Novartis, Roche, Sanofi, Takeda, Hikma, Celgene, Jansen, MSD, Abbvie, Pfizer and Amgen. Research support: Novartis, Roche, Takeda, Jansen, Pfizer and Amgen.

Vivian Blunk: Employee of and owner of stock in Pfizer.

### Author Contributions

All authors have contributed equally to the conception, design drafting, review, and finalization of manuscript.

## Appendices

**Appendix 1.** Search Strings

**Search strings in PubMed database**	
	
**African and Middle East** **("Multiple Myeloma"[Mesh] OR "Multiple myeloma") AND ("epidemiology" [Subheading] OR "prevalence" OR "incidence") AND ("Africa" OR "Middle East" OR "AfME" OR “MENA” OR “North Africa”) NOT ("case report") NOT ("consensus") NOT ("review")** **Selected countries of African and Middle East** **("Multiple Myeloma"[Mesh] OR "Multiple myeloma") AND ("epidemiology" [Subheading] OR "prevalence" OR "incidence") AND (“Iraq” OR “Lebanon” OR “Saudi Arabia” OR “UAE” OR “Algeria” OR “Egypt” OR “Morocco” OR “South Africa” OR “Jordan” OR “Kuwait” OR “North Africa” OR “Tunisia”) NOT ("case report") NOT ("consensus") NOT ("review")**	

	
**Treatment trends - Newly diagnosed MM** **African and Middle East** **("Multiple Myeloma"[Mesh] OR "Multiple myeloma") AND ("newly diagnosed" OR "new diagnosis") AND (“Africa” OR “Middle East” OR “AfME” OR “MENA” OR “North Africa”) NOT ("case report") NOT ("consensus") NOT ("review")** **Selected countries of African and Middle East** **("Multiple Myeloma"[Mesh] OR "Multiple myeloma") AND ("newly diagnosed" OR "new diagnosis") AND (“Iraq” OR “Lebanon” OR “Saudi Arabia” OR “UAE” OR “Algeria” OR “Egypt” OR “Morocco” OR “South Africa” OR “Jordan” OR “Kuwait” OR “North Africa” OR “Tunisia”) NOT ("consensus") NOT ("review")**	

**Treatment trends - Relapsed/Refractory MM** **African and Middle East** **relapsed [All Fields] OR refractory[All Fields] AND ("multiple myeloma"[MeSH Terms] OR multiple myeloma[Text Word]) AND (“Africa” OR “Middle East” OR “AfME” OR “MENA” OR “North Africa”) NOT ("case report") NOT ("consensus") NOT ("review")** **Selected countries of African and Middle East** **relapsed [All Fields] OR refractory[All Fields] AND ("multiple myeloma"[MeSH Terms] OR multiple myeloma[Text Word]) AND (“Iraq” OR “Lebanon” OR “Saudi Arabia” OR “UAE” OR “Algeria” OR “Egypt” OR “Morocco” OR “South Africa” OR “Jordan” OR “Kuwait” OR “North Africa” OR “Tunisia”) NOT ("case report") NOT ("consensus") NOT ("review")**	

**Treatment trends -Transplant-eligible MM patients** **African and Middle East** **"Transplant eligible" AND ("multiple myeloma"[MeSH Terms] OR multiple myeloma [Text Word]) AND (“Africa” OR “Middle East” OR “AfME” OR “MENA” OR “North Africa”) NOT ("case report") NOT ("consensus") NOT ("review")** **Selected countries of African and Middle East** **"Transplant eligible" AND ("multiple myeloma"[MeSH Terms] OR multiple myeloma [Text Word]) AND (“Iraq” OR “Lebanon” OR “Saudi Arabia” OR “UAE” OR “Algeria” OR “Egypt” OR “Morocco” OR “South Africa” OR “Jordan” OR “Kuwait” OR “North Africa” OR “Tunisia”) NOT ("case report") NOT ("consensus") NOT ("review")**	

**Treatment outcomes** **African and Middle East** **("multiple myeloma"[MeSH Terms] OR multiple myeloma [Text Word]) AND ("treatment outcome"[MeSH Terms] OR treatment outcome [Text Word]) AND (“Africa” OR “Middle East” OR “AfME” OR “MENA” OR “North Africa”) NOT ("case report") NOT ("consensus") NOT ("review")** **Selected countries of African and Middle East** **("multiple myeloma"[MeSH Terms] OR multiple myeloma [Text Word]) AND ("treatment outcome"[MeSH Terms] OR treatment outcome [Text Word]) AND (“Iraq” OR “Lebanon” OR “Saudi Arabia” OR “UAE” OR “Algeria” OR “Egypt” OR “Morocco” OR “South Africa” OR “Jordan” OR “Kuwait” OR “North Africa” OR “Tunisia”) NOT ("case report") NOT ("consensus") NOT ("review")**	

**Treatment outcomes – Mortality** **African and Middle East** **("multiple myeloma"[MeSH Terms] OR multiple myeloma [Text Word]) AND ("mortality"[Subheading] OR "mortality"[MeSH Terms] OR mortality [Text Word]) AND (“Africa” OR “Middle East” OR “AfME” OR “MENA” OR “North Africa”) NOT ("case report") NOT ("consensus") NOT ("review")** **Selected countries of African and Middle East** **("multiple myeloma"[MeSH Terms] OR multiple myeloma [Text Word]) AND ("mortality"[Subheading] OR "mortality"[MeSH Terms] OR mortality [Text Word]) AND (“Iraq” OR “Lebanon” OR “Saudi Arabia” OR “UAE” OR “Algeria” OR “Egypt” OR “Morocco” OR “South Africa” OR “Jordan” OR “Kuwait” OR “North Africa” OR “Tunisia”) NOT ("case report") NOT ("consensus") NOT ("review")**	

**Treatment outcomes – Survival** **African and Middle East** **("multiple myeloma"[MeSH Terms] OR multiple myeloma [Text Word]) AND ("mortality"[Subheading] OR "survival"[MeSH Terms] OR survival [Text Word]) AND (“Africa” OR “Middle East” OR “AfME” OR “MENA” OR “North Africa”) NOT ("case report") NOT ("consensus") NOT ("review")** **Selected countries of African and Middle East** **("multiple myeloma"[MeSH Terms] OR multiple myeloma [Text Word]) AND ("mortality"[Subheading] OR "survival"[MeSH Terms] OR survival [Text Word]) AND (“Iraq” OR “Lebanon” OR “Saudi Arabia” OR “UAE” OR “Algeria” OR “Egypt” OR “Morocco” OR “South Africa” OR “Jordan” OR “Kuwait” OR “North Africa” OR “Tunisia”) NOT ("case report") NOT ("consensus") NOT ("review")**	

**Appendix 2.** Characteristics of included studies

SL. No	Author, year	Study design	Sample size ()N	Type of MM	Median age	Male:Femlae	Treatment pattern	SES of country	Outcomes	Mortality	Follow-up period
1.	Novitzky N, 2010	Prospective study	42	Newly diagnosed MM	53 (33–68)	0.61	Induction–dexamethasone Sub-myeloablative doses of mel and stem cell support Sub-myeloablative dose transplant	South Africa UMIC	CR: 48% VGPR: 33% Progressive disease: 2% OS: 75%	8	648 days
2.	Kassem NM, 2014	Retrospective study	217	Newly diagnosed MM	53.5	1.4	Front-line therapy–endoxan and dexamethasone: 27 patients VAD regimen: 12 patients Thalidomide: 13 patients Velcade: 3 patients	Egypt LMIC	Median OS: 15.8 months	-	1–84 months
3.	Abdelgawad IA, 2016	Prospective study	89	Newly diagnosed MM	54 (45–59)	1.7	VAD OR VD	Egypt LMIC	At the end of the study Median OS: 74% Median DFS: 12 months		1–36 months
4.	Hammadi AMA, 2017	Prospective study	05	MM patients undergoing ASCT	50	4.0	ASCT with melphalan	Iraq UMIC	2 survived 2 relapsed 1 death	1	2–96 months
5.	Sallam Y, 2016	Prospective study	60	Newly diagnosed MM	55 (42–70)	1.5	**1st line:** 56.7%: VAD 28.3%: MP 5%: Dexamethasone Radiotherapy was given to 56.7% Bisphosphonates were given to 43.3% **2nd line:** 2.4%: CVP 2.4%: Dexamethasone–thalidomide 14.3%: VAD 6.50%: Bisphosphonates 2.4%: Dexamethasone **3rd line:** 25.9%: MP 22.2%: Dexamethasone 29.6%: Dexa–thalidomide 14.8%: DCEP 63%: Bisphosphonates 14.8%: Radiotherapy	Egypt LMIC	Median OS Patients <60 years: 37 months Patients <60 years: 12 months Median PFS: 6 months Patients with 1st line of treatment CR: 15.3% GPR: 11.9% PR: 6.8% Complications: 8.5% 2nd line of treatment CR: 2.5% GPR: 20% PD: 47.5% 3rd line of treatment PR: 19.2%		January 2005– December 2008
6.	Haleeqa MA, 2019	Retrospective study	62	-	43 (30–90)	2	-	UAE HIC	-	-	-
7.	Bedewy AML, 2020	Prospective study	33 MM patient, 15 healthy controls	Newly diagnosed MM	63 (51–75)	1.3	(Bortezomib and dexamethasone)	Egypt LMIC	Response to BD CR: 30.3% PR: 57.6% NR: 12.1%		Median 22.5 (12–34 months)
8.	Cowan A, 2020 Africa and East Mediterranean	Retrospective study	-	-	-	-	-	-	-	-	108 months
9.	Kassem NM, 2020	Prospective study	81	Newly diagnosed MM	53 (32–75)	0.93	All patients received bortezomib/cyclophosphamide/dexamethasone OR bortezomib/thalidomide/dexamethasone for 3-4 months Younger patients and high disease-burden patients received VRD Patients above 70 years: Endoxan/dexamethasone Maintained with lenalidomide–dexa	Egypt LMIC	Median OS VCD: 20.3 months VRD: 26.4 months VTD: 44.5 months Endoxan-dexa: 5.1 months CR: 16% PR: 39.5%	3	1–156 months
10.	Abdrabou AK, 2021	Retrospective study	169	Newly diagnosed MM	51 (23–69)	1.4	1. Induction–VAD After 2010 Induction– Thalidomide/dexa Bortezomib/thalidomide/dexa Cyclophosphamide/bortezomib/dexa 2. Mobilization–cyclophosphamide and G-CSF 3. High-dose chemotherapy 4. ASCT	Saudi Arabia HIC	Median OS post-transplant: 20 2 months Median PFS post-transplant: 30 months Pre-transplant CR: 38.1% PR: 38.1% VGPR: 11.3% Post-transplant CR: 57.4% PR: 6.5% VGPR: 11.8%	1	
11.	Bashaireh KM, 2021	Retrospective study	120	-	-	-	-	Jordan UMIC	-	-	2004–2018
12.	Fares S, 2021	Prospective study	64	Newly diagnosed MM	57 (30–65)	1.75	CTD VTD	Morocco LMIC	Median OS at 24 months: 83% Median PFS: 65.9%	8	25 months
13.	Kakoo A, 2021	Prospective study	32	29 newly diagnosed MM 03 relapsed MM	Mean: 64.5 (41–81)	1.4	Newly diagnosed: VTD/VD/VCD Relapsed MM: daratumumab (DARA) (all patients were aggressively treated prior to DARA)	Iraq UMIC	-	-	-
14.	Nasr F, 2021	Retrospective study	134	Newly diagnosed	Mean age: 61.91 ±10.83	1.6	Induction– VTD/VRD/VED/VER/VD/D/oncovin/ thalidomide/endoxan/RD/MPD/radiotherapy/ VAdriaDT/HyperCVAD/VDT+Alkeran/MP/ V+Caelyx+D Transplant–ASCT Consolidation Maintenance	Lebanon UMIC	Average OS: 52.57 months Significantly higher mean PFS in patients receiving transplants (75.11 months)	-	
15.	El Husseiny NM, 2013	Retrospective study	217	Newly diagnosed	Mean age: 58.5 (27–80)	1.4	Endoxan/steroids Melphalan/steroids Thalidomide (either alone or added to previous regimens), VAD regimen or Velcade	Egypt LMIC	Median OS: 37.5 months (1–84 months)	-	

ASCT: Autologous stem cell transplant; BD: Bortezomib-Dexamethasone; CR: Complete response; CTD: Cyclophosphamide-Thalidomide-Dexamethasone; CVAD: Cyclophosphamide-vortezomib-doxorubicin-dexamethasone; CVP: Cyclophosphamide-vincristine-prednisone; DCEP: Dexamethasone-cyclophosphamide-etopside-cisplatin; dexa: Dexamethasone; DFS: Disease free survival; G-CSF: Filgrastim; GPR: Good partial response; HIC: High income country; LMIC: Low and middle income country; Mel: Melphalan; MM: Multiple myeloma; MP: Melphalan-prednisone; MPD: Melphalan-prednisone-dexamethasone; NR: No response; OS: Overall survival; PD: progressive disease PFS: Progression-free survival; PR: Partial response; PVD: Pomalidomide-bortezomib-dexamethasone; RD: Lenalidomide-dexamethasone; RVD: lenalidomide-bortezomib-dexamethasone; UMIC: Upper middle income country; VAD: Vincristin-adriamycin-decadrone; VCD: Velcade-cyclophosphamide-dexamethasone; VD: Velcade-thalidomide; VED: Vincristine-epirubicin-dexamethasone; VGPR: Very good partial response; VRD: Velcade-lenalidomide-dexamethasone; VT: Velcade-thalidomide; VTD: Velcade-thalidomide-dexamethasone;
